# Synergistic adaptations: freezing tolerance is associated with desiccation tolerance and activation of violaxanthin de-epoxidase in wintergreen ferns

**DOI:** 10.1093/jxb/erab068

**Published:** 2021-04-02

**Authors:** Helen I Holmlund

**Affiliations:** Natural Science Division, Pepperdine University, Malibu, USA

**Keywords:** Desiccation tolerance, embolism, fern, freezing tolerance, *F*_v_/*F*_m_, wintergreen, xanthophyll, xylem

## Abstract

This article comments on:

**Fernández-Marín B, Arzac MI, López-Pozo M, Laza JM, Roach T, Stegner M, Neuner G, García-Plazaola JI**. 2021. Frozen in the dark: interplay of night-time activity of xanthophyll cycle, xylem attributes, and desiccation tolerance in fern resistance to winter. Journal of Experimental Botany **72**, 3168–3184.

**Some ferns can survive substantial abiotic stress. In this issue, Fernández-Marín *et al.* (2021*a*) identify an overlap between two seemingly disparate stressors: freezing and near-complete desiccation. The authors determine that three desiccation-tolerant (DT) fern species are also tolerant to freezing at –7 °C, analyzing several traits that may confer both freezing tolerance and desiccation tolerance. In particular, the authors discovered that DT ferns exhibit freezing-induced activation of violaxanthin de-epoxidase in the absence of light. This study complements previous studies finding overlap between desiccation tolerance and freezing tolerance in angiosperms and lichens, providing evidence for a broader trend across the phylogeny.**

Land plants have evolved diverse adaptations to the terrestrial environment, including tolerance of desiccation and freezing. DT (‘resurrection’) plants can dry to equilibrium with the air and resume metabolic activity upon rehydration (< –100 MPa; Alperts, 2006). DT plants are distinct from those which exhibit some degree of ‘dehydration tolerance’, a far more common trait across land plants (Oliver *et al.*, 2020; Box 1). In the fully desiccated state, DT plants lose nearly all of their free extracellular and intracellular water, forming a glassy state in which cellular components are stabilized by protective sugars and proteins (reviewed in Oliver *et al.*, 2020).

**Box 1. Desiccation tolerance versus dehydration tolerance**

Desiccation tolerance (DT) is not the same as tolerance of partial dehydration (DhT). DhT species can tolerate some degree of partial dehydration before suffering irreversible cellular damage. The degree of DhT is highly species dependent, but, to my knowledge, no DhT species can survive past –15 MPa, the point at which cellular processes are affected by biophysical changes in the membrane and cytosol (Oliver *et al.*, 2020). In contrast, DT plants can lose nearly all of their free water (<0.1 g H_2_O g^–1^ dry plant mass, or –100 MPa; Alperts, 2006). Loss of water occurs in an orchestrated sequence of events which leads to the desiccated, and virtually inactive, metabolic state. Upon rehydration, they rapidly (hours to days) regain metabolic activity (hence the term ‘resurrection’ plants). During freezing, plant cells are dehydrated, but it remains unclear whether freezing DT plant cells typically (i) suffer partial dehydration or (ii) transition into the desiccated, inactive state (but this could be species dependent, see Fernández-Marín et al., 2018; Georgieva *et al.*, 2020). A freezing-induced glassy state may explain the freezing tolerance observed in DT plants (Fernández-Marín et al., 2018), although further research is needed in this direction.

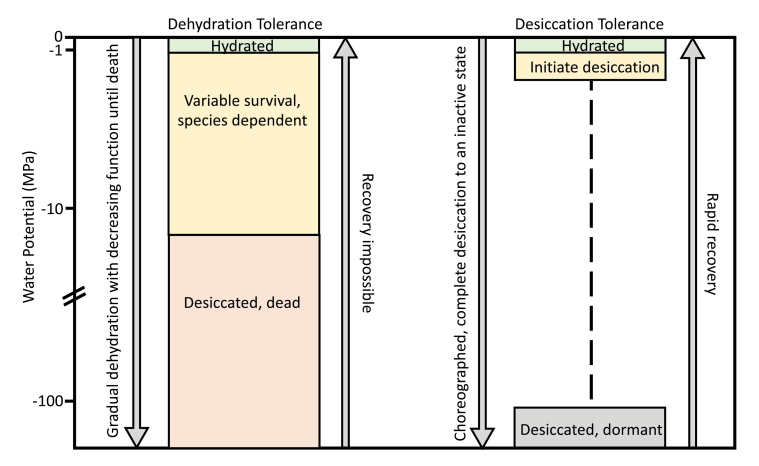

At surface level, the effects of freezing on plant tissues may seem distinct from the effects of severe dehydration leading to the desiccated state, but many plants experience some degree of cellular dehydration during freezing. The apoplastic water in xylem conduits is the first to freeze (Sakai and Larcher, 1987). When extracellular water freezes, water is pulled out of the living symplast, sometimes dehydrating the cells to <10% water content (Wolfe and Bryant, 1999). This state presents the typical hazards associated with severe dehydration (unstable cell membranes, reactive oxygen species, and photoinhibition) as well as those unique to freezing (ice crystals). With a further decrease in temperature, eventually the cell symplast can freeze, and the expansion of intracellular ice can critically disrupt cell membranes (Ball *et al.*, 2004).

## Similar adaptations to desiccation–rehydration and freeze–thaw events

Whole-plant tolerance of freezing and desiccation shares some similar mechanisms in both the symplast and the apoplast (Verhoeven *et al.*, 2018). In the symplast, Fernández-Marín *et al.* (2021*a*) identified some key similarities in DT ferns’ response to freezing and desiccation. In both cases, the maximum quantum efficiency of PSII (*F*_v_/*F*_m_) decreased in the frozen or desiccated state, and recovered to full capacity when this state was reversed. This decrease can be understood as controlled down-regulation of photochemical efficiency; that is, a photoprotective mechanism involving zeaxanthin. In parallel, violaxanthin de-epoxidase is activated during both the freezing and desiccating processes, converting violaxanthin to zeaxanthin, even in the absence of light (Fernández-Marín *et al.*, 2021*a*). These findings align with previous studies, suggesting a broader trend across DT photosynthetic organisms that tolerate freezing (Fernández-Marín et al., 2018, 2019, 2021*b*).

Resilience of the apoplast to desiccation and freezing is an important trait for vascular plants, including ferns and angiosperms. In the apoplast, desiccation and freeze–thaw events can both cause severe (excessive) accumulation of air embolism in the xylem conduits, blocking the flow of water through the plant vascular system. During desiccation, air embolisms are caused by increased xylem tension on the water column and subsequent air-seeding and cavitation (Jarbeau *et al.*, 1995). As severe dehydration continues, most of the residual free water in the walls of the xylem conduits evaporates, as bulk tissues reach a desiccated state. In freeze–thaw events, air is forced out of the water as it freezes, causing the air to coalesce into bubbles inside the xylem conduits (Langan *et al.*, 1997). As the tissue thaws, these bubbles are susceptible to expand under xylem tension, creating an air embolism (Davis *et al.*, 1999).

In both desiccation and freeze–thaw events, narrow xylem conduits confer an advantage. Following desiccation, small conduits aid in capillary rise during rehydration, facilitating whole-plant recovery (Sherwin *et al.*, 1998; Holmlund *et al.*, 2020). Narrow xylem conduits also confer increased freeze–thaw tolerance in the apoplast because small air bubbles formed during freezing are less likely to expand and cavitate during thaw (Davis *et al.*, 1999). Fernández-Marín *et al.* (2021a) noted this parallel adaptation in their study. Although all five fern species had narrow xylem conduits relative to species with known susceptibility to freeze–thaw embolism (<44 µm; Davis *et al.*, 1999; Pittermann and Sperry, 2003), the three DT species had a greater proportion of conductive area occupied by very narrow xylem conduits (<18 µm), supporting the hypothesis that narrow conduits are adaptive in DT plants (Fernández-Marín *et al.*, 2021a). Furthermore, it is likely that partial dehydration accompanied by a winter freeze can periodically interact to be a selective advantage for <18 µm xylem conduits (Langan *et al.*, 1997; Davis *et al.*, 2005).

Evidently, there is overlap between traits that confer tolerance of desiccation and freezing. Are all DT plants also freezing tolerant? So far, we have evidence that some DT plants are also tolerant of some degree of freezing (Fernández-Marín et al., 2018, 2019, 2020, 2021*b*). It is possible that all DT plants achieve a glassy state at sufficiently low temperatures, and this may confer cellular freezing tolerance. Vitrification during freezing has been suggested previously (Hirsh, 1987; Strimbeck *et al.*, 2015) and recently demonstrated in a DT angiosperm *Ramonda myconi* (Fernández-Marín et al., 2018). In their study on DT ferns, Fernández-Marín *et al.* (2021*a*) did not directly test for achievement of a desiccated glassy state or intracellular freezing in the symplast of DT ferns at –7 °C, but they discovered freezing-induced activation of violaxanthin de-epoxidase in the absence of light, which may be followed by more severe dehydration to achieve the glassy state. These results provide key insights into the mechanisms contributing to freezing tolerance in the symplast of DT plants. Perhaps the same mechanisms that initiate the glassy state in drying DT plant cells safeguard the symplast against intracellular dehydration during ice formation. If that is the case, then DT plants may survive very cold temperatures, with nearly all ice residing in the extracellular spaces and the cell symplast safely in the glassy state.

## The trade-off: maximizing carbon investment

Previous studies have observed that DT plants tend to be relatively small, suggesting a trade-off between desiccation tolerance and productivity (Alperts, 2006). Tolerance of desiccation and freezing both maximize the leaf carbon investment by maximizing assimilation at the beginning and end of the growing season (Alperts, 2006; Prats and Brodersen, 2020). DT and freezing-tolerant plants can resume photosynthesis within hours or days after these stressors abate, but this productivity gain also incurs costs. For example, narrow xylem conduits contribute to tolerance of desiccation and freezing, but this trait limits the hydraulic efficiency and corresponding growth rates when liquid water is abundant. Also, the glassy state requires a carbon investment of sugars and proteins to stabilize cellular components, diverting carbon that might have otherwise been used for growth.

What evolutionary pressures tip the scales in favor of desiccation or freezing tolerance? From an ecological perspective, these traits are likely to be favored in regions or microsites that experience frequent or lengthy desiccation or freezing. Habitats prone to both desiccation and freezing may select for tolerance to both desiccation and freezing. However, if freezing tolerance of DT plants is merely a mechanistic side effect of desiccation tolerance, then freezing tolerance in DT plants may be de-coupled from the minimum temperatures of their habitats. It would be interesting to test the freezing tolerance of DT epiphytes in subtropical regions, such as *Pleopeltis polypodioides* in south-eastern North America. Freezing tolerance in an organism that rarely experiences freezing could provide support for the hypothesis that the DT trait inherently confers some degree of freezing tolerance.

## Looking forward

Many questions remain about the intersection of desiccation tolerance and freezing tolerance. First, there are likely to be many DT plant species that have not yet been identified as such. Compiling lists of DT species is arduous work, partly because negative results are rarely published (researchers rarely find it surprising when dead plants stay dead). Furthermore, desiccation tolerance is often treated as a closed-ended question, with species identified positively or negatively as DT based on survival past –100 MPa, but there may be more complexity in the spectrum of DT plants. Techniques like the ‘Falcon test’ developed by López-Pozo et al. (2019) will probably aid surveys of DT plants and assessment of the range of plant DT.

In a similar way, much remains unknown about freezing tolerance, especially in ferns. Unlike desiccation tolerance, freezing tolerance is less often categorized as a closed-ended question, but rather it is generally expressed as a spectrum of tolerance, using metrics such as LT_50_ (temperature at which 50% of leaf cells die; Boorse *et al.*, 1998). Freezing may be an underappreciated driver of plant distribution in dry environments. For example, freeze–thaw events determine chaparral shrub distribution in southern California’s Mediterranean-type climate, a region also inhabited by resurrection plants (Davis *et al.*, 2007; Holmlund *et al.*, 2016). Consideration of the microsite is key because understorey plants may experience less radiation freeze than plants in a common garden without canopy protection from the cold night sky. Questions remain about the intersection of freezing and drought are particularly important in light of a changing climate, which could alter these stresses and species’ distributions.

## References

[CIT0001] Alpert P . 2006. Constraints of tolerance: why are desiccation-tolerant organisms so small or rare? Journal of Experimental Biology 209, 1575–1584. 10.1242/jeb.02179 16621938

[CIT0002] Ball MC , Canny MJ, Huang CX, Heady RD. 2004. Structural changes in acclimated and unacclimated leaves during freezing and thawing. Functional Plant Biology 31, 29–40. 32688878 10.1071/FP03164

[CIT0003] Boorse GC , Bosma TL, Meyer AC, Ewers FW, Davis SD. 1998. Comparative methods of estimating freezing temperatures and freezing injury in leaves of chaparral shrubs. International Journal of Plant Sciences 159, 513–521.

[CIT0004] Davis SD , Ewers FW, Pratt RB, Brown PL, Bowen TJ. 2005. Interactive effects of freezing and drought on long distance transport: a case study of chaparral shrubs of California. In: Holbrook NM, Zwieniecki MA, eds. Vascular transport in plants. Academic Press, 425–435.

[CIT0005] Davis SD , Helms AM, Heffner MS, Shaver AR, Deroulet AC, Stasiak NL, Vaughn SM, Leake CB, Lee HD, Sayegh ET. 2007. Chaparral zonation in the Santa Monica Mountains: the influence of freezing temperatures. Fremontia 35, 12–15.

[CIT0006] Davis SD , Sperry JS, Hacke UG. 1999. The relationship between xylem conduit diameter and cavitation caused by freezing. American Journal of Botany 86, 1367–1372. 10523278

[CIT0007] Fernández-Marín B , Arzac MI, López-Pozo M, Laza JM, Roach T, Stegner M, Neuner G, García-Plazaola JI. 2021 *a*. Frozen in the dark: interplay of night-time activity of xanthophyll cycle, xylem attributes, and desiccation tolerance in fern resistance to winter. Journal of Experimental Botany 72, 3168–3184. 33617637 10.1093/jxb/erab071

[CIT0008] Fernández-Marín B , López-Pozo M, Perera-Castro AV, et al 2019. Symbiosis at its limits: ecophysiological consequences of lichenization in the genus *Prasiola* in Antarctica. Annals of Botany 124, 1211–1226. 10.1093/aob/mcz149 PMC6943718 31549137

[CIT0009] Fernández-Marín B , Nadal M, Gago J, Fernie AR, López-Pozo M, Artetxe U, García-Plazaola JI, Verhoeven A. 2020. Born to revive: molecular and physiological mechanisms of double tolerance in a paleotropical and resurrection plant. New Phytologist 226, 741–759. 10.1111/nph.16464 32017123

[CIT0010] Fernández-Marín B , Neuner G, Kuprian E, Laza JM, García-Plazaola JI, Verhoeven A. 2018. First evidence of freezing tolerance in a resurrection plant: insights into molecular mobility and zeaxanthin synthesis in the dark. Physiologia Plantarum 163, 472–489. 29345751 10.1111/ppl.12694

[CIT0011] Fernandez-Marin B , Roach T, Verhoeven A, Garcia-Plazaola J. 2021 *b*. Shedding light on the dark side of xanthophyll cycles. New Phytologist, doi:10.1111/nph.17191 33452715

[CIT0012] Georgieva K , Mihailova G, Velitchkova M, Popova A. 2020. Recovery of photosynthetic activity of resurrection plant *Haberlea rhodopensis* from drought- and freezing-induced desiccation. Photosynthetica 58, 911–921.

[CIT0013] Hirsh AG . 1987. Vitrification in plants as a natural form of cryoprotection. Cryobiology 24, 214–228. 3595165 10.1016/0011-2240(87)90024-1

[CIT0014] Holmlund HI , Davis SD, Ewers FW, Aguirre NM, Sapes G, Sala A, Pittermann J. 2020. Positive root pressure is critical for whole-plant desiccation recovery in two species of terrestrial resurrection ferns. Journal of Experimental Botany 71, 1139–1150. 31641748 10.1093/jxb/erz472 PMC6977189

[CIT0015] Holmlund HI , Lekson VM, Gillespie BM, Nakamatsu NA, Burns AM, Sauer KE, Pittermann J, Davis SD. 2016. Seasonal changes in tissue-water relations for eight species of ferns during historic drought in California. American Journal of Botany 103, 1607–1617. 27638918 10.3732/ajb.1600167

[CIT0016] Jarbeau JA , Ewers FW, Davis SD. 1995. The mechanism of water-stress-induced embolism in two species of chaparral shrubs. Plant, Cell & Environment 18, 189–196.

[CIT0017] Langan SJ , Ewers FW, Davis SD. 1997. Xylem dysfunction caused by water stress and freezing in two species of co-occurring chaparral shrubs. Plant, Cell & Environment 20, 425–437.

[CIT0018] López-Pozo M , Flexas J, Gulías J, et al 2019. A field portable method for the semi-quantitative estimation of dehydration tolerance of photosynthetic tissues across distantly related land plants. Physiologia Plantarum 167, 540–555. 30515832 10.1111/ppl.12890

[CIT0019] Oliver MJ , Farrant JM, Hilhorst HW, Mundree S, Williams B, Bewley JD. 2020. Desiccation tolerance: avoiding cellular damage during drying and rehydration. Annual Review of Plant Biology 71, 7.1–7.26. 10.1146/annurev-arplant-071219-105542 32040342

[CIT0020] Pittermann J , Sperry J. 2003. Tracheid diameter is the key trait determining the extent of freezing-induced embolism in conifers. Tree Physiology 23, 907–914. 14532014 10.1093/treephys/23.13.907

[CIT0021] Prats KA , Brodersen CR. 2020. Seasonal coordination of leaf hydraulics and gas exchange in a wintergreen fern. AoB Plants 12, doi: 10.1093/aobpla/plaa048 PMC7724977 33324481

[CIT0022] Sakai A , Larcher W. 1987. Frost survival of plants: responses and adaptation to freezing stress. Berlin Heidelberg: Springer Science & Business Media.

[CIT0023] Sherwin HW , Pammenter NW, February ED, Vander Willigen C, Farrant JM. 1998. Xylem hydraulic characteristics, water relations and wood anatomy of the resurrection plant *Myrothamnus flabellifolius* Welw. Annals of Botany 81, 567–575.

[CIT0024] Strimbeck GR , Schaberg PG, Fossdal CG, Schröder WP, Kjellsen TD. 2015. Extreme low temperature tolerance in woody plants. Frontiers in Plant Science 6, 884. 26539202 10.3389/fpls.2015.00884 PMC4609829

[CIT0025] Verhoeven A , García-Plazaola JI, Fernández-Marín B. 2018. Shared mechanisms of photoprotection in photosynthetic organisms tolerant to desiccation or to low temperature. Environmental and Experimental Botany 154, 66–79.

[CIT0026] Wolfe J , Bryant G. 1999. Freezing, drying, and/or vitrification of membrane–solute–water systems. Cryobiology 39, 103–129. 10529304 10.1006/cryo.1999.2195

